# T2-Mapping CMR increases the diagnostic sensitivity for detection of myocardial inflammation in patients with possible myocarditis - a prospective endomyocardial biopsy-controlled study

**DOI:** 10.1186/1532-429X-17-S1-P319

**Published:** 2015-02-03

**Authors:** Florian Bönner, Maximilian Spieker, Beate Stanske, Sebastian M Haberkorn, Britta Butzbach, Patrick Horn, Felix T Range, Ulrich Flögel, Mirja Neizel-Wittke, Malte Kelm, Ralf Westenfeld

**Affiliations:** 1 University Düsseldorf, Düsseldorf, Germany

## Background

Myocarditis has been reported in up to 20% of sudden cardiac death in young adults and is a frequent precursor of dilated cardiomyopathy. Unfortunately, the diagnostic tools for detection of myocarditis are still imperfect: Sensitivity of endomyocardial biopsy (EMB) is reduced largely due to the inherent sampling error. Cardiac magnetic resonance (CMR) offers the advantage of analysing the whole myocardium, but contrast-enhanced as well as T2-weighted CMR exhibit inadequate sensitivity, especially during early stages of inflammation.

Our hypothesis was that quantitative T2 relaxation mapping increases diagnostic sensitivity in CMR-based diagnosis of myocarditis.

## Methods

We carried out a prospective observational study in patients with probable acute myocarditis characterized by clinical presentation, new global or regional wall abnormalities or arrhythmias or hsTNT-elevation. Of the 55 patients screened, two patients did not undergo CMR (1 pacemaker, 1 ECLS-support) and 16 patients refused EMB. The remaining 37 patients underwent EMB and CMR examination (1.5 T, Archieva, Philips) within 36h. Histological evaluation was performed by two independent pathologists (hematoxylin eosin staining, picrosirius red, IH CD68, CD45R0 and CD3) and by molecular analysis for viral replication/genome. CMR data were analysed blinded with respect to ventricular volumes and ejection fraction as well as T2, LGE and Strain Encoded (SENC)-Imaging. A GRASE sequence (15 Echoes separated by 10ms, res: 2x2x10 mm2, 3 short axis slices) was used for localized T2 mapping. Age-matched volunteers (37) served as controls for ROC curve analysis in terms of quantitative T2-mapping. Results were compared by two-sided t-Test; p<0.05 was considered significant.

## Results

In the 37 patients with "probable myocarditis" undergoing EMB+CMR, 26 patients were identified as "biopsy-proven myocarditis" (70%). To apply quantitative analysis strategies we determined the mean physiological T2 relaxation in healthy, age matched controls and performed ROC-curve analysis compared to patients with biopsy-proven myocarditis. We determined that a threshold of 3% myocardium with absolute T2 times above 80ms detected patients with "biopsy-proven myocarditis" with a sensitivity 0,93 and a specificity of 0,96. Larger areas of myocardium with pathological T2 times correlated significantly with reduced LV-function (*p<0.05). Applying this technique to patients with "probable myocarditis", a diagnosis of myocarditis was made in 34 (92%) of the patients. Among all patients, 60% were characterized as myocarditis on the basis of LGE and only 9% on the basis of T2 Imaging alone. There was no significant correlation of local myocardial systolic strain with elevated T2 values.

## Conclusions

Conventional CMR and EMB are of additive diagnostic value in patients with possible myocarditis. However, T2 mapping facilitates generation of objective, quantitative criteria for the diagnosis of myocarditis and enhances the sensitivity in CMR-based diagnosis.

## Funding

None.

